# Rheumatism

**Published:** 1856-01

**Authors:** E. S. S. Rouse

**Affiliations:** Mount Vernon, O.


We published, in a former number, a poem written by our poetic friend in which he indulged in certain denunciations against the disease from which, it would appear, he has been happily relieved. It would, we think, be a difficult question to determine to which of the remedies he can attribute the relief experienced, those detailed below, or the *mercurial* and *drastic* effects of his severe denunciations.

---

**RHEUMATISM.**

BY E. S. S. ROUSE.

The Poet having got relief, publisheth the recipe for the benefit of other folks, and the rest of mankind.

In my late severe affliction, when, regardless of restriction,
I did vent my malediction, from behind my chamber door;
As you saw, in Coggeshall's paper* 'twas not all an idle vapor,
As one cuts a foolish caper, just for fun, and nothing more;
Nothing more:—

But a truthful lamentation of my dreadful situation,
And the fearful aggravation of the racking pains I bore.
Was'nt I a dog that's lucky,—from the town of Keokuk-ee,
Which has recently been stuck-ee on the Mississippi shore,
Nothing more;—

From that place, so fresh and vernal, issued forth the Medic Journal,
Be its name and fame eternal, and for aye and evermore:—
For in its human pity, it took up my dismal ditty,
As a tale both sad and witty, and demanding an encore,
Nothing more.

And now from its ample pages, the M. D.'s of future ages,
May surmise the awful stages of the dread disease I bore,
By the awful execrations, and the fell denunciations,
And the fearful imprecations inadvertently I swore;
(Nothing more.)

Now I'll write out the prescription which hath cured without deception,
Those pains beyond description, of which I wrote before;
So you men of medic science, may feel a firm reliance,
In bidding them defiance, henceforth forevermore;—
Evermore.

'Ply a poultice made of mustard, little thicker than a custard,
Like you'd feed a chicken bustard (but that is meal, and nothing more,)
If you want it any hotter, tell your pretty little daughter,
Just to bring some scalding water, and to pour it slowly o'er;—
Nothing more!

---

* See *Genius of the West*, for February, 1855: also *The Iowa Medical Journal*, for April and May, 1855.

Let your mother or your sister have a care it doesn't blister,
And your wife may well assist her, if there's need of any more;
Lest in very deed and double, when you come to burst the bubble,
It should prove a source of trouble, and become a running sore,
And nothing more.

Or perhaps 'twill cure you faster of your terrible disaster,
If you take a sticking plaster,—P. Burg. and something more;
And then take—videlicit—Tart. Ant. quant. sufficit,
(Since I have to be explicit,) and sprinkle it well o'er;
Nothing more.

Apply it to the part affected;—if you do as I've directed,
Then a cure may be expected in a month, if not before,
Then, altho' you're much my debtor, if you've followed the strict letter,
And very fast have got no better,—you may *try something more!*
SOMETHING MORE ! †

MOUNT VERNON, O. Sept. 11th, 1855.

† The Poet hath the characteristic of the true physician; who, when one *infallible* remedy faileth, recommendeth to "*try, try again*

